# Zinc-Containing Surgical Stents for Soft Tissue Healing: Clinical Case Series and Chair-Side Application

**DOI:** 10.3390/reports9020111

**Published:** 2026-04-02

**Authors:** Blagovesta Yaneva, Dobromira Shopova, Liliya Kavlakova, Georgi Boychev, Petar Shentov, Atanaska Dinkova

**Affiliations:** 1 Department of Periodontology and Oral Diseases, Faculty of Dental Medicine, Medical University of Plovdiv, 4000 Plovdiv, Bulgaria; liliya.kavlakova@mu-plovdiv.bg (L.K.); georgi.boychev@mu-plovdiv.bg (G.B.); petar.shentov@mu-plovdiv.bg (P.S.); 2 Laser and Physical Dental Medicine, Research Institute, Medical University of Plovdiv, 4000 Plovdiv, Bulgaria; atanaska.dinkova@mu-plovdiv.bg; 3 Department of Prosthetic Dentistry, Faculty of Dental Medicine, Medical University of Plovdiv, 4000 Plovdiv, Bulgaria; 4 CAD/CAM Center, Research Institute, Medical University of Plovdiv, 4000 Plovdiv, Bulgaria; 5 Department of Oral Surgery, Faculty of Dental Medicine, Medical University of Plovdiv, 4000 Plovdiv, Bulgaria

**Keywords:** zinc, zinc-based surgical stents, periodontal disease, soft tissue regeneration, wound healing, biocompatibility, case report

## Abstract

**Background and Clinical Significance**: The optimization of soft tissue healing following oral surgical procedures remains a key factor for achieving long-term functional and esthetic success. This article aims to explore the clinical application and healing potential of zinc-containing stents in the management of various oral soft tissue conditions. **Case Presentation**: Four clinical cases involving different etiologies of soft tissue lesions were included: (1) persistent pregnancy-associated gingival enlargement, (2) prosthesis-related gingival inflammation, (3) plaque-induced gingivitis, and (4) palatal thermal injury.Zinc-containing stents were fabricated from preheated granulate and applied following initial or supportive plaque control. Patients were instructed to wear the stents for a prescribed period. Clinical parameters, including the full mouth plaque score (FMPS), full mouth bleeding score (FMBS), tissue appearance, and patient comfort, were evaluated during follow-up. All four patients demonstrated complete resolution of clinical signs, including reduced inflammation, improved gingival contour, and accelerated tissue healing, without reported discomfort or adverse effects. In inflammatory cases, FMPS and FMBS values decreased markedly after stent use, while the palatal burn lesion showed complete re-epithelialization within five days. No adverse effects or complications were observed during follow-up periods ranging from one week to one year for the different cases. **Conclusions**: Zinc-containing stents show promising clinical potential as adjunctive tools in the management of periodontal and oral mucosal conditions. Their bioactive properties—anti-inflammatory, antimicrobial, and regenerative—may enhance soft tissue healing and patient comfort. Further controlled clinical studies are needed to establish standardized treatment protocols and optimize zinc formulations for wider adoption in clinical practice.

## 1. Introduction and Clinical Significance

Diseases of the oral mucosa comprise a broad and diverse group of pathological conditions affecting the soft tissues of the oral cavity, including the lips, cheeks, gingiva, tongue, palate, and the floor of the mouth. These disorders vary in etiology and clinical manifestation, encompassing infectious, inflammatory, autoimmune, systemic, traumatic, and neoplastic origins. Accurate recognition and diagnosis are essential for effective management and prevention of complications [1,2].

Infectious diseases of the oral mucosa represent a significant portion of oral pathology and are caused by a wide variety of microbial agents, including bacteria, viruses, fungi, and, less commonly, protozoa. The oral cavity provides a favorable environment for microbial colonization due to its warm, moist conditions and constant exposure to external agents. These infections may arise primarily within the mouth or secondary as manifestations of systemic diseases. Their clinical presentation varies widely, from mild transient lesions to severe, disseminated conditions, depending on the etiologic agent and the host’s immune response [3,4,5,6].

Inflammatory diseases of the oral mucosa represent a diverse group of disorders characterized by immune-mediated or nonspecific inflammatory reactions affecting the lining of the mouth. These conditions vary in etiology, clinical presentation, and severity, but they commonly manifest as erythema, edema, ulceration, or desquamation of the mucosal tissues. The inflammatory process may be acute or chronic and can arise as a result of local irritation, infection, hypersensitivity, or autoimmune mechanisms [7,8,9].

Traumatic and reactive lesions of the oral mucosa constitute a common group of conditions that develop as a response to various forms of physical, chemical, or thermal injury. These lesions represent the tissue’s adaptive or reparative reaction to chronic irritation, acute trauma, or repetitive mechanical stress. Although generally benign and non-neoplastic, their clinical appearance may mimic pathological entities of greater concern, making accurate diagnosis essential [10,11,12].

A number of systemic diseases may manifest with oral mucosal changes, serving as early indicators of underlying pathology. Recognition of these manifestations is crucial for early detection and management of systemic conditions [13,14,15].

Potentially malignant and malignant lesions of the oral mucosa constitute a critical area in oral pathology, given their potential for progression to oral squamous cell carcinoma (OSCC), the most prevalent malignancy of the oral cavity. Early recognition, accurate diagnosis, and prompt management of such lesions are of utmost importance to improve prognosis and patient survival. These conditions often begin as localized epithelial changes that may appear clinically subtle but histologically reveal dysplastic transformation, indicating an increased risk of malignancy [16,17,18,19].

Soft tissue management is a crucial aspect of contemporary oral surgery and implantology, influencing both the biological integration and the esthetic outcome of treatment. Rapid epithelialization, minimal inflammation, and controlled healing architecture are desired for predictable results. Various techniques and materials have been proposed to support these processes, including collagen matrices, platelet concentrates, and customized healing abutments [20,21,22].

Zinc (Zn) plays a significant role in tissue regeneration due to its anti-inflammatory, antioxidant, and antimicrobial effects, as well as its involvement in collagen synthesis and epithelial repair. Incorporating Zn into chair-side fabricated surgical stents presents a simple and cost-effective approach to improve wound protection, stabilization of the blood clot, and modulation of the local healing environment. The bacteriostatic effect withholds bacteria from proliferating in the oral cavity: on the tooth surface and soft tissue such as gingiva, palate, and tongue. The Zn-containing granulate protects the wound, which can aid the healing process and can have a positive effect on patient comfort and observed pain [23,24,25]. Although zinc is recognized for its biological properties, few studies explore its incorporation into office-fabricated surgical grafts for soft tissue protection and healing. This case series addresses this gap.

This study aims to evaluate clinical efficacy, biocompatibility, and therapeutic benefits of zinc-oxide-enriched surgical stents in diverse oral soft tissue conditions, including inflammatory, reactive, and traumatic lesions.

## 2. Case Presentation 

This case series includes patients undergoing epulis excision, gingival inflammation, severe gingivitis and soft tissue burning, where soft tissue protection was preferable. Patients are above 18 years old, with no systemic diseases or no systemic medications that could influence the healing processes. Exclusion criteria for the application of zinc-containing surgical stents were pregnancy, immunocompromised patients, and zinc allergy. All procedures were performed in accordance with the Declaration of Helsinki. Written informed consent was obtained from all patients for the treatment and publication of anonymized clinical data and images, and ethical approval was obtained. Chair-side Zn-containing surgical stents were fabricated from a polymer enriched with zinc oxide nanoparticles (Perio Plast, Elemental, Limburg, Belgium). After placing the granulate in hot water (80 °C) the material becomes mouldable and can be manually shaped to cover the desired area in the oral cavity. The design was adapted individually to cover the surgical site, ensuring mechanical protection, optimal marginal adaptation, and comfort.

Clinical parameters including pain, edema, epithelialization rate, and soft tissue color were evaluated postoperatively.

**Figure 1:**
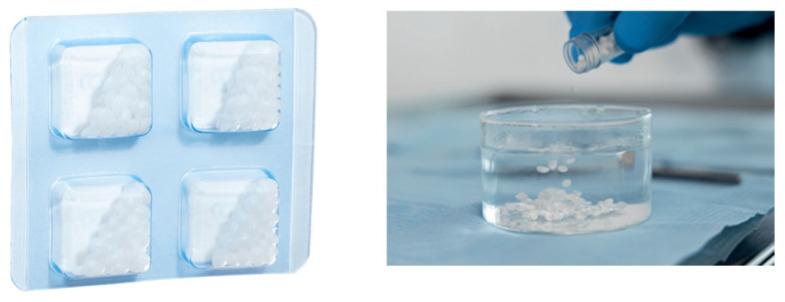
Zn-containing granulate for surgical stent [https://www.withelemental.com/en-eu].

The Zn-containing stent, Figure 1, is a dental granulate made from a bacteriostatic polymer, which, after preheating, can be fitted around the desired site in the oral cavity.

### 2.1. Case 1

**Figure 2:**
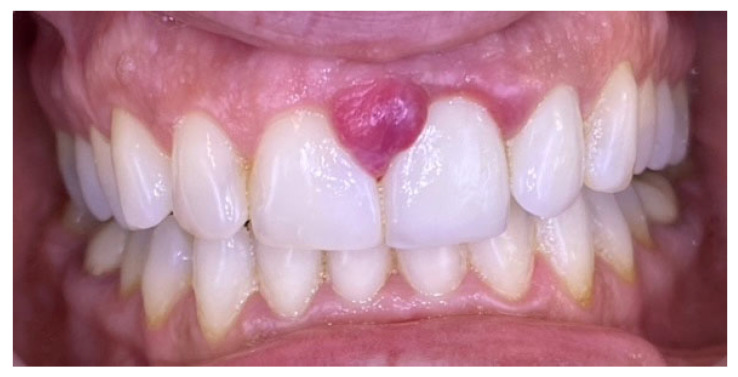
Case 1. Gingival progenic granuloma between the central incisors of the upper jaw. Generalized gingival inflammation.

A 36-year-old female presented with complaints of gingival enlargement, bleeding on provocation, and an unaesthetic appearance. The patient presented no systemic diseases or medications. She is a nonsmoker. Clinical examination revealed a tumor-like enlargement of the interdental papilla between the upper central incisors, classified as score I according to Angelopoulos and Goaz classification of gingival enlargement (Figure 2). Teeth 21 and 22 were restored with zirconium crowns. The lesion developed during pregnancy but did not regress one year after childbirth. The patient demonstrated poor oral hygiene (full mouth plaque score/FMPS: 68%) and generalized gingivitis (full mouth bleeding score/FMBS: 56%).

Initial professional mechanical plaque control using an ultrasonic scaler and air-flow device (EMS Prophylaxis Master, Nyon, Switzerland) was performed, and oral hygiene instructions were given. After four weeks, FMPS and FMBS decreased significantly to 18% and 15%, respectively, but the gingival enlargement persisted.

**Figure 3:**
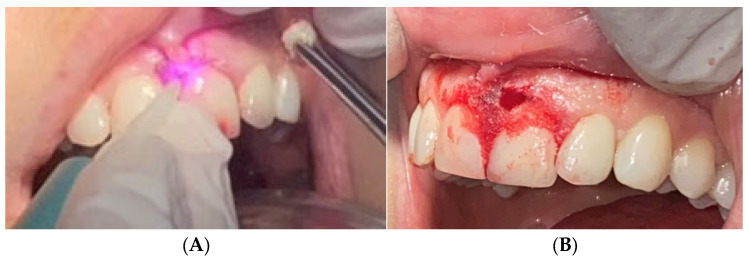
Case 1—Laser-assisted excisional biopsy of gingival progenic granuloma (**A**) with 980 nm diode laser with 300 µm fiber diameter. (**B**) Immediately post-operative appearance with hemostasis achieved.

**Figure 4:**
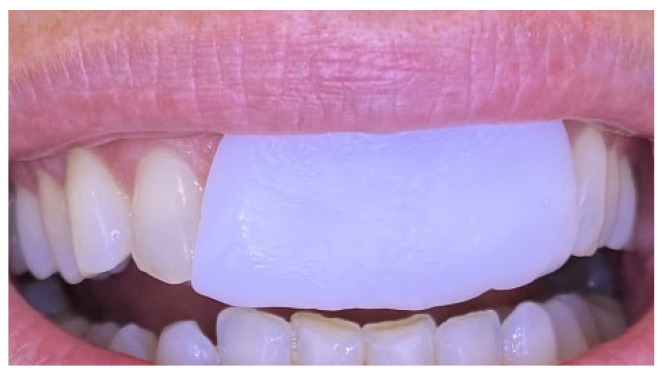
Case 1. Zinc-containing stent applied over the surgical site.

**Figure 5:**
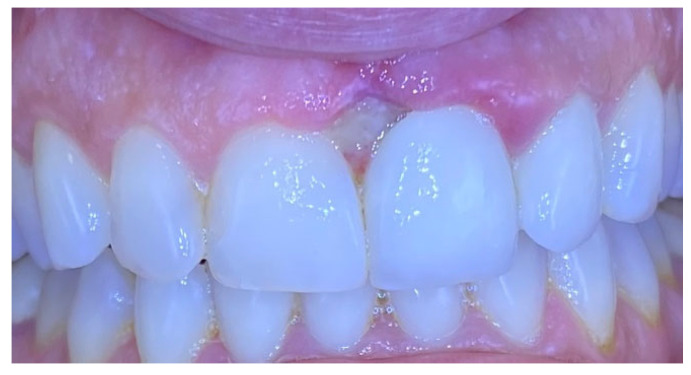
Case 1. Surgical site 3 days after surgery.

**Figure 6:**
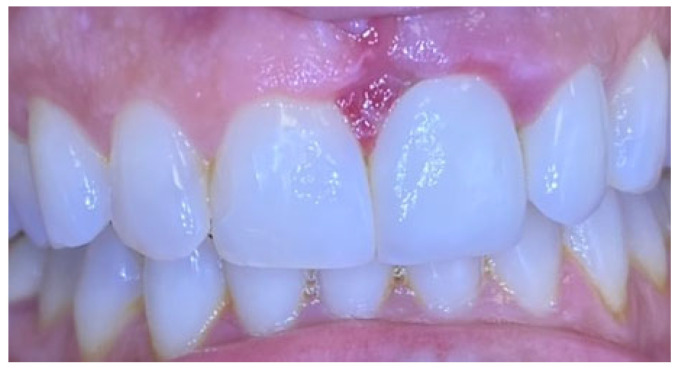
Case 1. Interdental papilla 1 week after surgery.

**Figure 7:**
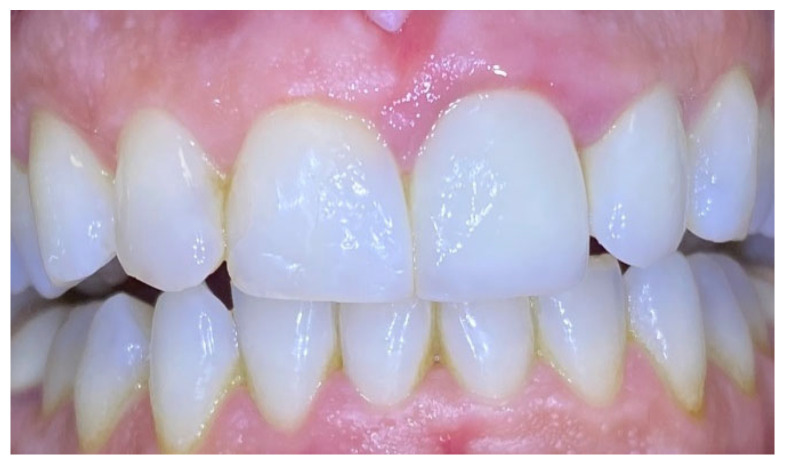
Case 1. Interdental papilla 1 month after surgery.

**Figure 8:**
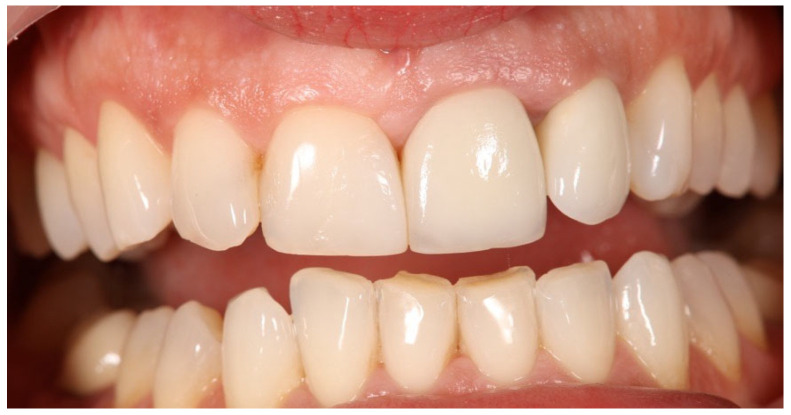
Case 1. Interdental papilla between upper central insicors 2 years after surgery.

Excisional biopsy of the lesion was carried out with a diode laser (980 nm wavelength, 300 µm fiber diameter; Lite Medics, Italy) (Figure 3). The surgical site was covered with a zinc-containing stent prepared from preheated granulate (Figure 4). Histopathological examination confirmed the diagnosis of gingival pyogenic granuloma. The patient wore the stent for one week, 8 h daily. Healing went uneventfully and was satisfactory. The patient reported no swelling and bleeding, limited pain in the first 48 h and no discomfort afterwards. The stent protected the surgical site and provided faster re-epithelization. The patient is enrolled in a periodontal maintenance program with control visits every 4 months. No recurrence was observed after 2 years of follow-up (Figure 5, Figure 6, Figure 7 and Figure 8).

### 2.2. Case 2

**Figure 9:**
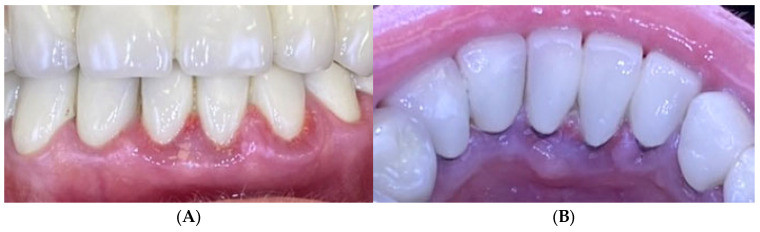
Case 2. Gingival inflammation around the lower frontal teeth baseline. (**A**)—Vestibular view; (**B**)—lingual view.

A 68-year-old female presented with gingival bleeding following placement of a fixed prosthesis in the lower arch two weeks earlier. She reported no systemic diseases or medication use. Clinical examination revealed a partially edentulous lower arch with both fixed and removable prostheses. Gingival inflammation and enlargement were noted around the lower anterior teeth (Figure 9). Oral hygiene assessment revealed FMPS of 65% and FMBS of 73%.

**Figure 10:**
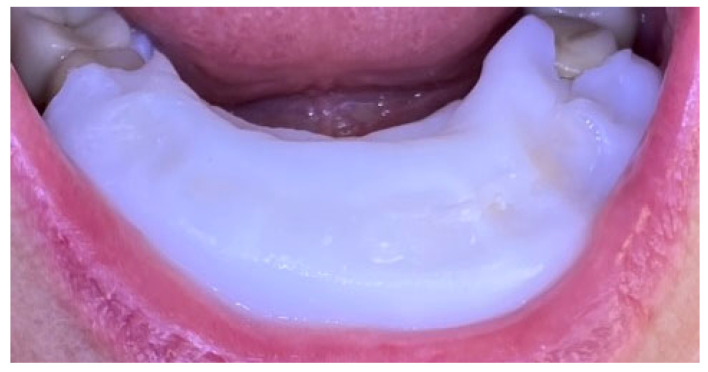
Case 2. Zinc-containing stent placed over the lower frontal teeth and gingival tissues.

The patient received oral hygiene instruction and professional plaque removal using ultrasonic and air-flow devices. A zinc-containing stent was fabricated from preheated granulate to cover the lower anterior region (Figure 10), and the patient was instructed to wear it as much as possible during day and night.

**Figure 11:**
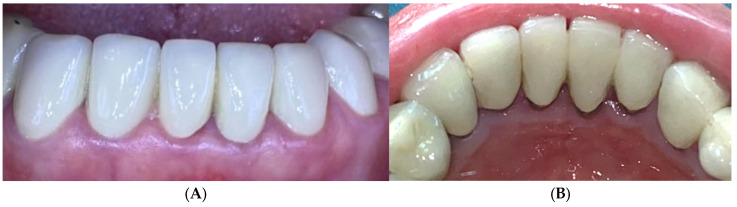
Case 2. One month after application of zinc-containing stent over the lower frontal teeth. (**A**)—Vestibular view of the gingival tissues; (**B**)—lingual view of the gingival tissues.

After one week, FMPS and FMBS improved to 19% and 25%, respectively, with a marked reduction in gingival inflammation. After wearing the zinc-containing stent, the color of the gingiva was coral pink and the swelling reduced dramatically. At the one-month follow-up, no signs of gingival inflammation were observed, gingival health was achieved, the patient stopped wearing the stent and was included in maintenance periodontal therapy with recall visits every 3 months (Figure 11).

### 2.3. Case 3

**Figure 12:**
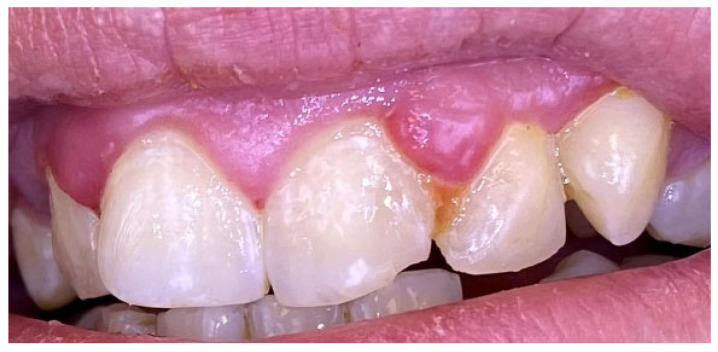
Case 3. Severe gingivitis of upper jaw before treatment.

A 25-year-old male presented with bleeding gums. Oral examination revealed poor oral hygiene, with gingival erythema and edema. FMPS was 85% and FMBS was 78%. A zinc-containing stent fabricated from preheated granulate was applied to cover the upper arch (Figure 12). The patient was instructed to wear the stent at least 8 h per day for 1 week.

**Figure 13:**
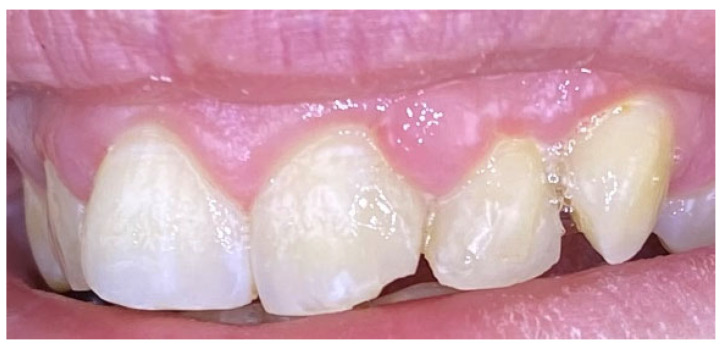
Case 3. Gingival tissues 1 week after application of zinc-containing stent without professional cleaning.

After one week of use, gingival inflammation had decreased significantly—the swelling of the gingiva decreased significantly as well as the color of the free gingival margin and interdental papilla. The FMBS improved to 56%, reflecting clear clinical resolution of inflammation (Figure 13). No side effects or patient discomfort was reported during the use of the stent. Afterwards, professional mechanical plaque control using ultrasonic and air-polishing devices was performed, and gingival health was established (FMBS—10% and FMPS—15%). The patient was included in maintenance periodontal therapy with follow-up visits every 6 months.

### 2.4. Case 4

**Figure 14:**
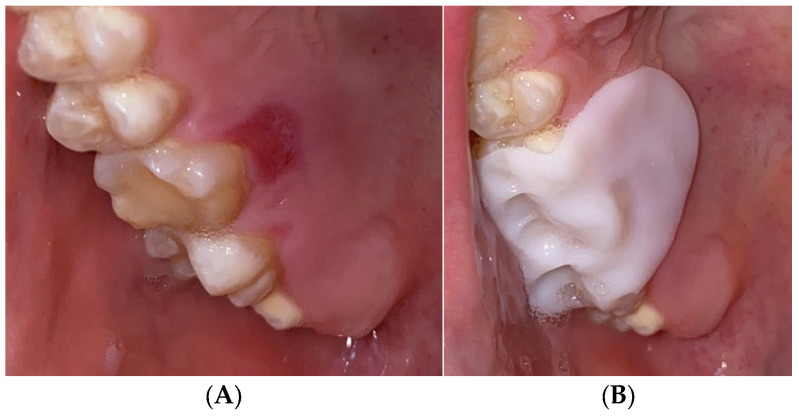
Case 4. (**A**) Baseline. Thermal injury of the hard palate after consuming hot food. (**B**) Adaptation and application of zinc-containing stent over the traumatic ulcer.

A 22-year-old male presented with severe palatal pain following consumption of extremely hot food. Clinical examination revealed an erosive lesion on the right side of the palate in the region of tooth 16. A zinc-containing stent was prepared from preheated granulate and applied to cover the affected area (Figure 14). The patient was instructed to wear the stent as much as possible during the day and night.

**Figure 15:**
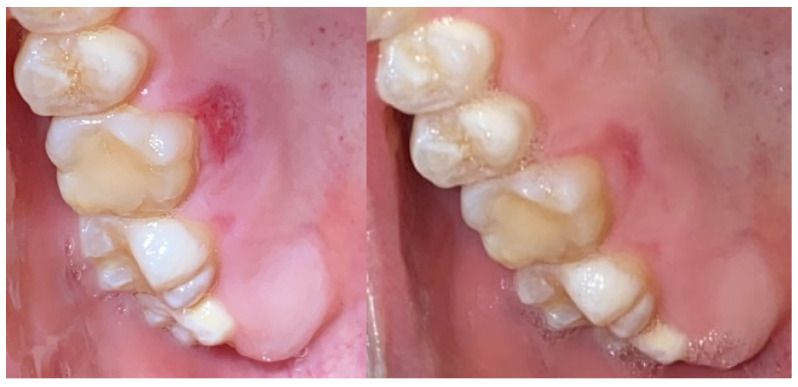
Case 4. Healing of the thermal injury in 2 and 5 days.

Complete healing of the lesion was observed within five days. The patient reported an uneventful healing period with a decrease in symptoms, no side effects or adverse reactions (Figure 15).

Summary of findings is presented in Table 1:

## 3. Discussion

Diseases of the oral mucosa encompass a wide spectrum of pathological conditions with diverse etiologies and clinical manifestations. Their proper identification requires a comprehensive understanding of oral pathology, careful clinical examination, and, when necessary, histopathological confirmation. As the oral cavity often reflects systemic health, the clinician’s awareness of mucosal changes plays a vital role in both local disease management and the early detection of systemic disorders and malignancies [1,5,8,13,18].

The stents used in this study are Zn-containing biomaterials; however, the manufacturer does not disclose the exact quantitative composition. Therefore, the discussion of zinc-related effects in this manuscript are based on previously published literature describing the biological properties of Zn in comparable biomedical applications.

Zinc ion release kinetics and local bioavailability: Zinc oxide nanoparticles primarily contribute potent antimicrobial activity through the release of Zn2+ ions, disrupting bacterial cell membranes and biofilm architecture, with additional anti-inflammatory effects reported in gingival tissues [26].

Comparative advantages of zinc-containing stents vs. conventional wound dressings are a stable microenvironment (stents offer greater structural stability and prolonged function), lower bacterial colonization, ease of fabrication chair-side, and mechanical stability [27,28].

### 3.1. Case 1

The present case illustrates the successful management of a persistent pregnancy-associated gingival enlargement through surgical excision supported by a zinc-containing stent for enhanced soft tissue healing. Gingival enlargement associated with pregnancy is a common reactive lesion influenced by hormonal changes that exacerbate the local inflammatory response to dental plaque. In most cases, regression occurs following childbirth; however, persistence may occur due to fibrotic transformation or continued plaque accumulation [29,30].

Initial nonsurgical therapy significantly reduced plaque accumulation and gingival bleeding (FMPS: 68% to 18%; FMBS: 56% to 15%), indicating good patient compliance and improvement in general periodontal condition. Nonetheless, the persistence of localized enlargement suggested a chronic inflammatory or fibrotic lesion, confirmed histopathologically as gingival pyogenic granuloma. Surgical excision was, therefore, indicated to eliminate the residual lesion and restore gingival contour. The use of a diode laser provided a minimally invasive surgical approach with superior hemostasis, reduced postoperative pain, and enhanced patient comfort compared with conventional scalpel excision. Following excision, the surgical site was covered with a zinc-containing stent designed to stabilize the wound and promote tissue healing [31,32,33].

Zinc plays a crucial role in numerous biological processes, including cell proliferation, epithelial repair, and immune regulation. It is an essential cofactor for matrix metalloproteinases and superoxide dismutase, both of which are involved in collagen synthesis and oxidative stress modulation [25,34]. The anti-inflammatory and antimicrobial properties of zinc ions have been shown to enhance epithelial regeneration and reduce bacterial colonization in wound areas. These characteristics support the hypothesis that zinc-containing biomaterials can accelerate mucosal healing following surgical interventions in the oral cavity [35,36].

In this case, the patient demonstrated satisfactory healing without discomfort or complications, and no recurrence was observed during a two-year follow-up. The results suggest that zinc-containing stents may serve as a valuable adjunct in oral soft tissue management, particularly in cases requiring enhanced healing and postoperative protection.

### 3.2. Case 2

This case highlights the effective management of prosthesis-induced gingival inflammation using a zinc-containing stent as an adjunct to nonsurgical periodontal therapy. Gingival enlargement and bleeding following prosthetic rehabilitation are frequently associated with plaque retention at restoration margins and inadequate oral hygiene [37,38]. In the present case, the patient demonstrated poor oral hygiene, as reflected by elevated FMPS (65%) and FMBS (73%) scores, indicating a high inflammatory burden. The adaptation of the crown edges of new prosthetic constructions, as well as parafunctions, can lead to reactive reactions from the periodontium [39,40,41].

Initial therapy focused on mechanical plaque removal and oral hygiene reinforcement, which led to a substantial improvement in clinical parameters after one week (FMPS: 19%; FMBS: 25%). However, the adjunctive use of a zinc-containing stent likely enhanced healing outcomes and patient comfort. By providing a protective physical barrier and maintaining a moist microenvironment, the stent may have minimized mechanical irritation from the prosthetic components and facilitated gingival tissue recovery.

Zinc is well recognized for its multifaceted role in soft tissue repair. It exerts anti-inflammatory, antimicrobial, and antioxidant effects, contributing to the modulation of cytokine release and the promotion of fibroblast proliferation [33,34]. Moreover, zinc supports collagen synthesis and epithelialization—key processes in mucosal healing [24,25]. In biomaterial applications, zinc-containing formulations have demonstrated the ability to accelerate wound closure and reduce infection risk, making them suitable for intraoral applications where microbial control is critical [23,42].

The complete resolution of gingival inflammation within one month, without recurrence or adverse effects, underscores the potential of zinc-containing stents as an adjunctive tool in managing prosthesis-related soft tissue complications. Beyond their local healing benefits, these biomaterials may also enhance patient compliance by improving comfort and protection during the healing phase.

### 3.3. Case 3

This case illustrates the effective adjunctive use of a zinc-containing stent in the initial management of generalized gingival inflammation associated with poor oral hygiene. Gingival bleeding, erythema, and edema are hallmark clinical signs of plaque-induced gingivitis, a reversible inflammatory condition resulting from the accumulation of bacterial biofilm at the gingival margin [37,38]. The patient presented with high plaque and bleeding scores (FMPS: 85%; FMBS: 78%), confirming a significant level of local inflammation.

The application of a zinc-containing stent for one week led to a notable reduction in bleeding and inflammation (FMBS decreased to 56%), suggesting that the stent contributed positively to soft tissue recovery even before professional debridement. This improvement may be attributed to both mechanical protection—by shielding the gingiva from further irritation—and biochemical activity related to zinc ion release [35,36,42].

Following professional plaque removal, gingival health was fully reestablished, confirming the reversibility of the condition and highlighting the combined effectiveness of mechanical and biomaterial-based interventions. The results from this case indicate that zinc-containing stents may serve as a valuable transitional aid in patients with acute gingival inflammation—particularly when immediate mechanical therapy is contraindicated or delayed.

### 3.4. Case 4

This case demonstrates the successful use of a zinc-containing stent in the management of a palatal mucosal burn resulting from thermal injury. Thermal trauma to the oral mucosa is relatively common and is typically caused by the consumption of excessively hot food or beverages. Such injuries can lead to epithelial necrosis, acute pain, and increased susceptibility to secondary infection [43,44]. Conventional management is usually symptomatic, involving topical agents or palliative dressings; however, these measures often provide limited protection and do not actively promote tissue repair.

In the present case, the application of a zinc-containing stent provided immediate mechanical protection of the injured area and contributed to rapid tissue recovery. Complete re-epithelialization occurred within five days, and the patient reported no pain or discomfort during the healing period, indicating that the stent successfully maintained a favorable wound environment.

Zinc’s bioactivity is well documented in wound healing. It supports keratinocyte migration, collagen synthesis, and epithelial proliferation, while exerting anti-inflammatory and antimicrobial effects that reduce the risk of infection [45,46]. In addition, zinc modulates oxidative stress and enhances tissue regeneration by regulating metallothionein and matrix metalloproteinase activity [47]. These properties likely contributed to the accelerated healing and absence of discomfort observed in this case. The zinc-containing stent functioned as both a protective dressing and a bioactive delivery system, combining physical shielding with localized biochemical support for tissue regeneration. Such dual action represents an advancement over conventional wound management in the oral cavity, where maintaining an undisturbed and moist environment is challenging due to salivary flow and mechanical forces [48].

The favorable outcome in this case supports the potential role of zinc-containing biomaterials in managing minor oral injuries and mucosal defects. Future investigations should assess their comparative performance against standard protective dressings and explore optimized formulations for broader clinical use in oral mucosal healing.

The presented cases collectively demonstrate the clinical potential of zinc-containing stents as effective adjuncts for managing various oral soft tissue conditions, including inflammatory lesions, prosthesis-related gingival enlargements, acute gingivitis, and thermal injuries. In all cases, the application of zinc-containing stents resulted in accelerated healing, reduced inflammation, and improved patient comfort without any adverse effects or recurrence.

## 4. Conclusions

Zinc’s potent anti-inflammatory, antimicrobial, antioxidant, and regenerative properties drive enhanced healing. These stents deliver both mechanical protection and bioactive support, creating a stable environment for efficient tissue repair.

Based on these cases, zinc-containing stents may be considered as an adjunctive tool for: (1) post-surgical protection after laser-assisted excision, (2) management of prosthesis-related gingival inflammation, (3) rapid resolution of acute gingivitis, and (4) emergency treatment of thermal injuries to the oral mucosa.

Zinc-containing stents emerge as promising bioactive tools in modern periodontal and oral surgery, particularly in cases demanding accelerated healing and reliable postoperative protection. Future studies should establish standardized protocols, define optimal zinc concentrations, assess long-term biocompatibility (>6 months), and compare zinc stents with conventional materials to establish evidence-based recommendations.

## Figures and Tables

**Table 1:** Summary of Clinical Cases and Outcomes.

	Patient Demographics	Diagnosis	Baseline Scores	Final Scores	Healing Time	Follow-Up
**Case 1**	36-year-old female	Gingival progenic granuloma	FMPS 68% FMBS 56%	FMPS 18% FMBS 15%	7 days	Every 4 months
**Case 2**	68-year-old female	Plaque-induced gingivitis	FMPS 65% FMBS 73%	FMPS 19% FMBS 25%	1 month	Every 4 months
**Case 3**	25-year-old male	Plaque-induced gingivitis	FMPS 85% FMBS 78%	FMPS 15% FMBS 10%	1 month	Every 6 months
**Case 4**	22-year-old male	Thermal traumatic ulcer	-	-	5 days	Every 6 months

## Data Availability

Data are available upon request from the authors. The data are not publicly available due to privacy concerns.

## Abbreviations

The following abbreviations are used in this manuscript:

OSCC	Oral Squamous Cell Carcinoma
Zn	Zinc
FMPS	Full Mouth Plaque Score
FMBS	Full Mouth Bleeding Score
